# Supplementary material for: Fabrication, Crystalline Behavior, Mechanical Property and In-Vivo Degradation of Poly(l–lactide) (PLLA)–Magnesium Oxide Whiskers (MgO) Nano Composites Prepared by In-Situ Polymerization
Source: Polymers (Basel). 2019 Jul 2;11(7):1123. doi: 10.3390/polym11071123 (PMC6680788; doi:10.3390/polym11071123)
Supplement: Supplementary file 1 [file polymers-11-01123-s001.pdf]

The preparation of composite film

The synthesized sample is fully dissolved in dichloromethane solution and then it was poured into a culture dish of 80 mm diameter, and then placed in a fume hood, after the dichloromethane were volatilized completely and the composite films were obtained.

The preparation of the standard dumbbell specimen

The standard dumb bell specimen was prepared by obtaining the films and dried at 60 °C for 24 h, and the film was punched into a dumb bell shape using with a punch machine, and the electronic micro-tension machine was controlled by MTF-100 microcomputer. The tensile test was carried out at a tensile speed of 0.1 mm/min, and three parallel samples were taken for each of the films.

The measurement of molecular weight (M_η)

The viscosity-average molecular weights (M_η) of the neat PLLA and nanocomposites were identified by viscometric measurements using an Ubbelohde Capillary Viscometer type 1835 (0.3–0.4 μm). The process preparation are as follows: At the first step, the implanted samples were dissolved by chloroform to prepare a solution with concentration of 0.01 g/ml and then dilute to 0.0096, 0.0092, and 0.0088 g/ml. Before charging the sample solution directly into the viscometer, the solution should be filtered using hydrophilic membrane filter of 0.45 micron size. In the second step, the solution with various concentration were put into ukrainian-style viscometer, respectively. Meanwhile, ukrainian-style viscometer was warmed in a constant temperature bath (25 ± 0.5 °C) for 15 min and the measurement was started with solvent until for five consecutive values which has been differ from their mean value by not more than 0.3 s. Further, flow times of five different concentrations of sample solution were recorded. The corrected average flow time for a solvent and each PLLA concentration, ti(corr was calculated by subtracting the Hagenbach correction, $\Delta t_{HC}(i)$ from the average flow time for that concentration, t_i, according to $t_i(corr=t_i-\Delta t_{HC}(i)$.

For each samples concentration, the following viscosities were determined using the following equation: η_{rel} = t/t_s(relative viscosity), $\eta_{sp}=(t/t_s)-1$ (specific viscosity), η_{red} = ηsp/c (reduced viscosity), η_{inh} = ln η_{red}/C (inherent viscosity). The reduced viscosity and inherent viscosity were plotted against chitosan concentration. The value of viscosity can be calculated by extrapolating graph of reduced viscosity and inherent viscosity to zero concentration. The average of the obtained intercept values were been calculated. This value was calculated from $[\eta]=KM_\eta\alpha$ equation, where $K=11.2 \times 10^{-4}$ (dm^3/g) and $\alpha=0.73$ determined in chloroform and PLLA solution at 25 °C.

NMR

The typical ^1H NMR spectra of the neat PLLA and the PLLA1 are shown in supplementary Figure S1, and the molecular structural of PLLA is shown in supplementary scheme S1. The major peaks of pure PLLA located at 1.56 and 5.15 ppm were assigned to CH and CH_3,[1] while the calculated average chemical shift for the Hα protons in the conformer of L-LA pentamer was 5.29 ppm, which agrees with the experimental value observed from the polymer. As from the observation, the CH peak at 1.57 ppm of PLLA1 were split into three distinct peaks was different from that of neat PLLA. This change might be caused due to the formation of intermolecular H-bonding between carbonyl groups and –OH groups from MgO or the PLLA chains polymerized on the MgO surface. However, the –OH or COOH groups associated with CH_3, CH, and C=O groups were not observed because of a relatively long

chain impact.[2] The result of ^{13}C NMR analysis is shown in supplementary Figure S2, the peak centered at 77 ppm was corresponding to the solvent CDCl3, the carbon peaks of PLLA assigned to the CH_3 (16.85 ppm), CH (69.21 ppm) and C=O (169.81 ppm) groups were observed, in comparison, it is similar to the spectrum of PLLA1, because no isomerism was generated between PLLA and MgO. Meanwhile, some of the chain end groups were hardly detected in the carbon spectrum since long chain impact of PLLA.[3]

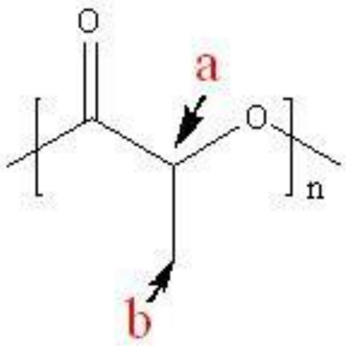

Scheme S1. Molecular structural formula of PLLA.

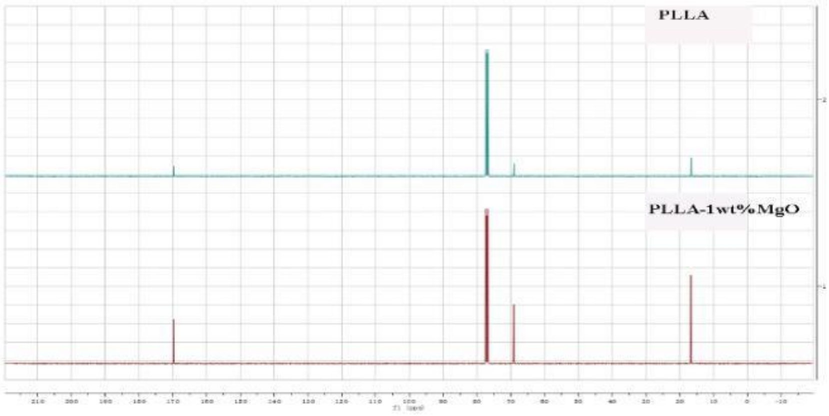

Figure S1. The 1H NMR spectra of PLLA and PLLA1

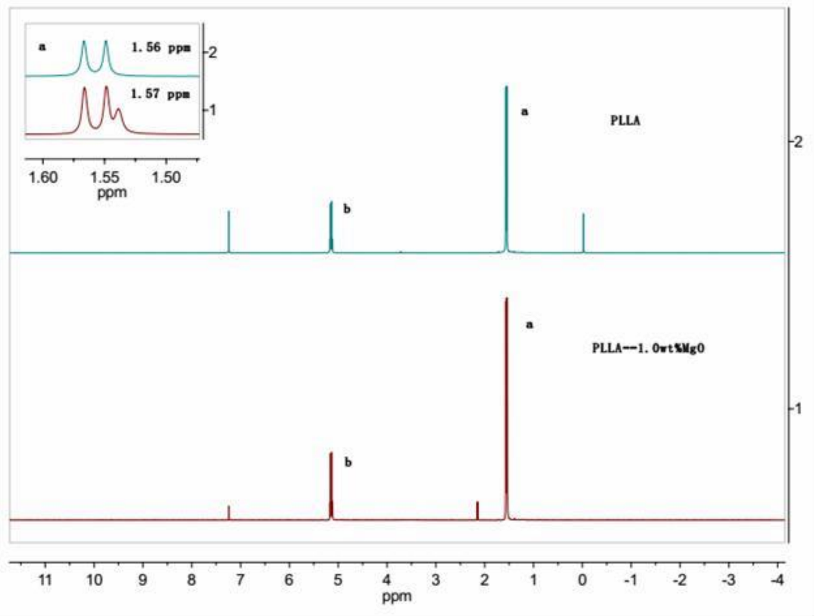

Figure S2. The ^{13}C NMR spectra of PLLA and PLLA1

Reference

1. Kum, C.H.; Cho, Y.; Seo, S.H.; Joung, Y.K.; Choi, J.; Park, K.; Park, Y.K.; Ahn, D.J.; Han, D.K. Biodegradable poly(L-lactide) composites by oligolactide-grafted magnesium hydroxide for mechanical reinforcement and reduced inflammation. *Small* **2013**, *1*, 2764–2772.

2. Chang, H.K.; Cho, Y.J.; Joung, Y.K.; Ahn, D.J.; Han, D.K. Poly(lactide) stereocomplex structure with modified magnesium oxide and its effects in enhancing the mechanical properties and suppressing inflammation. *Small* **2014**, 10, 3783-3794.

3. Luo, Y.B.; Wang, X.L.; Wang, Y.Z. Effect of nucleation and plasticization on the crystallization of poly (lactic acid). *Polym. Degrad. Stab* **2012**, 97, 721-728.
